# Design of Multifunctional Composites: New Strategy to Save Energy and Improve Mechanical Performance

**DOI:** 10.3390/nano10112285

**Published:** 2020-11-18

**Authors:** Liberata Guadagno, Andrea Sorrentino, Patrick Delprat, Luigi Vertuccio

**Affiliations:** 1 Department of Industrial Engineering, University of Salerno, Via Giovanni Paolo II, 84084 Fisciano, SA, Italy; 2 Institute for Polymers, Composites, and Biomaterials (IPCB-CNR), via Previati n. 1/E, 23900 Lecco, Italy; andrea.sorrentino@cnr.it; 3 ARKEMA S.R.L, BP 34, 64170 Lacq, France; delprat@arkema.com

**Keywords:** nanocomposites, electrical properties, cure behavior, joule effect, electro-curing

## Abstract

In this paper, an alternative curing strategy, based on the application of an electric field, is proposed to harden nano-filled multifunctional resins. The resin is obtained through the dispersion of carbon nanotubes, which act as nanometric heater elements in the epoxy matrix. The electro-curing is activated by applying an external electric voltage, which allows tunable cross-linking within the epoxy matrix entrapped between the nanotubes. The electro-curing method allows reaching higher curing degrees with respect to the conventional ones and, consequently, higher glass transition temperatures. This is a direct consequence of the fact that the curing reactions start directly in the regions at the interphase between carbon nanotubes, acting as heater nano-filaments, and the polymeric matrix. The proposed method is able to give composites better properties, making the curing process fast and energy-saving.

## 1. Introduction

Composite materials modified with nanoparticle incorporation manifest high performances in different functional and structural properties, which make them more advantageous than conventional materials in many sectors, among which are automotive, infrastructure, and aeronautic applications, etc. [1,2,3,4]. Nanostructured forms of carbon can be incorporated in epoxy resin to confer self-sensing properties, anti/de-icing ability, auto-repair function, adhesive properties, durability, etc. [5,6,7,8,9,10,11,12]. Furthermore, the past decade has witnessed an increasingly massive application of innovative composites for complex structural parts and components of aircrafts, cars, building structures, etc. [13,14]. The curing process of epoxy composites is currently based almost exclusively on thermal curing cycles, for which an oven or an autoclave is used [15]. This kind of curing, besides being an energy- and time-consuming process, requires wasteful costs for infrastructure [16], most of all when carbon nanotubes (CNTs) are incorporated in the matrix. Moreover, the low thermal conductivity of unfilled polymeric resins strongly limits the dimensions and geometries that can be employed during the manufacturing processes [17]. High-temperature gradients can induce non-homogeneous polymerizations, with the consequent formation of unbalanced internal shrinkage and stress [15,18]. Alternative curing methods for composite manufactures are of great industrial interest and can help save energy. Several criteria, such as material, operating conditions (temperature, humidity, mechanical stress, etc.), curing/heating speed, know-how, handling complexity, and cost, must be considered to make the right choice. If the curing speed and energy-saving issues are considered, the irradiation hardening process provides a technological superiority with respect to thermal hardening [19,20]. However, this process requires expensive radiation-sensitive resins. They must be transparent to the radiation and especially stable to environmental UV [19,21]. Heating with infrared and laser radiation presents the problem of the “surface heating” mechanism, while microwave radiation is limited by the nature of coupling with carbon fiber composites [15]. High-energy radiation sources, such as gamma rays, X-rays, and electron beams, can provide deep penetration into polymers and composites, but they are very expensive and manifest poor application flexibility due to their radioactive nature [22,23]. New curing methods based on different principles are being developed more and more. For instance, the use of carbon fibers as electrical resistors has been suggested in some aeronautical composites [24,25]. The cure was promoted by the heat produced through the current flowing in the fibers [26]. The heating homogeneity of this solution is generally poor as it depends on the fibers’ distribution along the composite. An alternative approach, based on the incorporation of nanometric fillers in the matrix, has been recently proposed [27,28]. When adequately dispersed, these fillers are able to form an electrical percolation network in the matrix, even at very low values of weight fraction [29,30]. Furthermore, the incorporation of nanometric fillers improves the thermal conductivity of the material [9,10,31,32,33]. The polymerization of resin under the application of an electrode potential is an alternative method. In this case, the cure reaction is electrochemically induced by an electron transfer reaction [34]. The process can be activated on-demand by applying a voltage potential that “switches on” the functional groups responsible for the cross-link reaction. The course of the reactions can be controlled by tuning the applied voltage [34,35]. It allows the end-user to modify the final material properties. The main drawback of this method is that the material curing takes place directly on the surface of the electrode where the electro-generation of the initiators is responsible for starting the polymerization of the monomers. The curing reaction is, thus, limited to the volume sample close to the working electrode and cannot propagate in a large sample [36,37,38]. Ping et al. [39] developed a method for curing on-demand adhesive through low-voltage electro-curing. They synthesized a grafting carbene precursor on polyamidoamine dendrimers and used it as the precursor for an instant curing adhesive. With low voltage application (−2 V versus Ag/AgCl), the carbene groups cross-link electrode surfaces and neighboring dendrimers, allowing to tune the material properties and the adhesion strength [39]. In this paper, an alternative curing process based on the electro-curing process has been proposed. The distribution of CNTs in the formulated samples was found to be satisfactory at the micrometric scale. The temperature increase generated by the CNTs subjected to the electric field was used to activate the cure reaction in the whole sample. The results were compared with those obtained with the same resin traditionally cured in an oven. Understanding the effect of the processing conditions is valuable for improving and/or optimizing the application of this strategy in several fields, such as adhesives, coatings, structural joints, and additive manufacturing.

## 2. Materials and Methods

### 2.1. Materials and Sample Preparation

**Figure 1:**
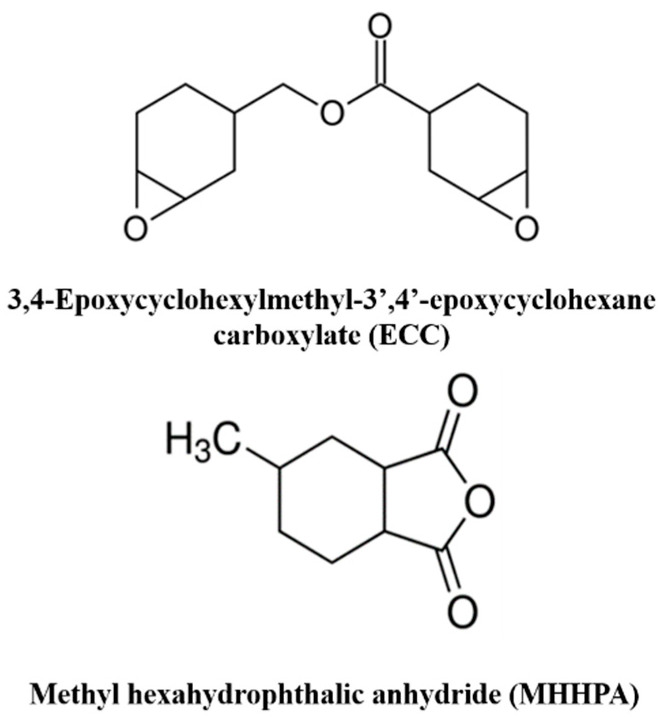
Structural formulas of the compounds.

The epoxy resin studied in this work consisted of a 3,4-epoxycyclohexylmethyl-3′,4′-epoxycyclohexane carboxylate (ECC) and a methyl hexahydrophthalic anhydride (MHHPA) as curing agents. They were supplied by Gurit (Italy) and used without any modifications. The molecular structures of the resin and the curing agents are shown in Figure 1. Carbon nanotubes (CNTs) were supplied by ARKEMA (GRAPHISTRENGTH C100). Thermogravimetric analysis (TGA) showed a carbon purity higher than 90% and a weight loss at 105 °C of less than 1%. A detailed analysis of their morphology was performed using high-resolution transmission electron microscopy (HR-TEM). The CNTs were characterized by an external diameter between 10 and 15 nm, a length between 0.1 and 10 µm, and a number of walls varying between 5 to 15.

**Figure 2:**
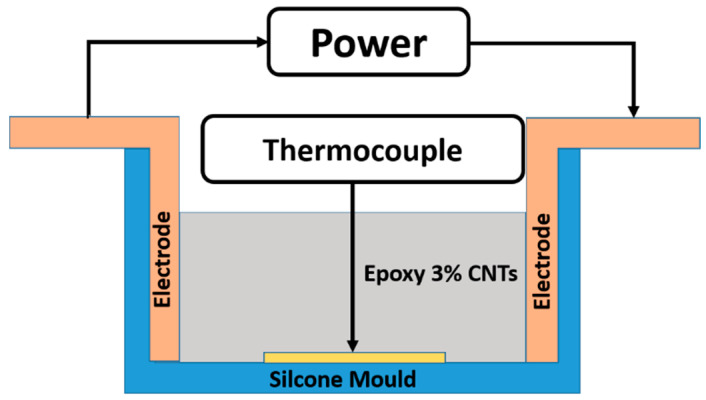
Scheme of the set-up used.

In order to obtain a uniform dispersion, the as-received CNTs were embedded into the precursor (at 3% weight of the final mixture) using an ultrasonic device for 20 min (Hielscher model UP200S-24 kHz high power ultrasonic probe). The hardener was added to the filler/precursor mixture with a ratio by weight of 1/1. The obtained mixture was mixed for 20 min at room temperature by magnetic stirring and then poured into a silicone mold with a size of 50 × 25 × 8 mm^3^. Samples were degassed for 2 h at room temperature and then cured in two different modes: a conventional heating cycle and an electro-curing process. For the heating cycle, the sample was transferred to an oven, which was set at 80 °C and maintained at this temperature for 60 min (acronym UF1 sample). The oven temperature was then increased up to 120 °C at 5 °C/min and then held at that value for 20 min (acronym UF2 sample). After that, the UF2 sample was post-cured at 180 °C for 60 min to complete the conversion of epoxy groups (acronym UF3 sample) and then the samples were left to cool down at room temperature. This curing procedure was selected as a reliable method to perform a comparison between the effect of the traditional curing method, based on the heating in the oven, and that of the electro-curing process on the final properties of the samples [40,41]. For the electro-curing, the silicone molds were equipped with two copper electrodes with a thickness of 80 μm (Figure 2). The electrodes were connected to a power supply EA-PSI 8360-10T (Elektro-Automatik, 0–360 V, 0–10 A, 1000 W max). A data acquisition board (Thermocouple Data Logger supplied by Pico Technology) was used to acquire the thermocouple measurements. The temperature of the mixture was controlled by a thin wire thermocouple with negligible thermal inertia (Type K Omega Engineering Ltd. Norwalk, CT, USA), which was positioned at the center of the mold (Figure 2).

### 2.2. Characterization Methods

The electrical tests of the samples were carried out by an HP 34401A multimeter (Keithley) in a 2-wire configuration. All tests were performed by applying a direct current (DC) with an HP E3631A 80 W Triple Output Power Supply (Keithley). The volumetric electrical resistivity was measured according to the Cabot Test Method (CTM) E043 based on ASTM D4496. The dimensions of the cured samples were 40 × 10 × 4 mm. As contacts, two silver paint electrodes, painted to completely cover the ends of the specimens, were used. After applying a DC voltage between the electrodes, the electrical conductivity (*σ*) of the specimen was calculated from the following Equation (1):

(1) $$\sigma =\frac{L}{A\times R}$$

where *R* is the electrical resistance; *A* is the cross-sectional area of the specimen; and *L* is the distance between the electrodes. The contact resistance has been considered negligible since the measured electrical resistance was in the order of kΩ [42,43]. Differential scanning calorimetry (DSC) was carried out by a thermal analyzer Mettler DSC 822/400 (Mettler-Toledo) purged with nitrogen. The area below the enthalpic curves was used to evaluate the curing degree (D.C.). The following Equation (2) was used:

(2) $$C.D.=100\times \left(\frac{\Delta H_{Dyn-}\Delta H_{residual}}{\Delta H_{Dyn}}\right)$$

where Δ*H_dyn_* is the total heat of reaction and Δ*H_residual_* is the residual heat of reaction obtained after the curing cycle (in the oven or by electro-curing). Several temperature cycles were investigated to evaluate the degree of cure of the epoxy system (1:1 precursor/hardener weight ratio). Water absorption into the samples was followed through mass uptake measurements. The samples were completely immersed in water at a constant temperature of 60 °C. At fixed periods, the samples were removed from the medium and wiped using a dry cloth before immediately weighing. The absorbed moisture concentration (*C*(*t*)) was calculated as:

(3) $$C\left(t\right)=\frac{W\left(t\right)-W\left(0\right)}{W\left(0\right)}$$

where *W*(*t*) and *W*(0) are the actual and initial sample weight, respectively. The dynamic mechanical measurements of the developed materials were carried out using a dynamic thermo-mechanical analyzer (TA instrument-DMA 2980, New Castle, DE, USA). During the tests, the samples (35 × 10 × 4 mm^3^) were subjected to a variable flexural deformation. The displacement amplitude was fixed to 0.02 mm, and the frequency to a value of 1 Hz in the interval of temperatures between −60 and 260 °C, setting a scanning rate of 3 °C/min. Transmission electron microscopy (TEM) characterization was performed on a JEOL 2010 LaB6 microscope operating at 200 kV. Thin slices were cut from the cured samples using an ultra-microtome equipped with a diamond knife.

SEM micrographs of the samples were obtained by the SEM LEO 1525 (Carl Zeiss SMT AG, Oberkochen, Germany). The cured samples were fractured in liquid nitrogen and then etched using an oxidizing solution before sputter coating. The oxidizing solution was used to etch the epoxy phase and increase the contrast between the CNT and the epoxy matrix [44]. Etched samples were placed on an aluminum stub and covered with a 250-Å thick gold film by using a sputter coater (Agar mod. 108 A).

## 3. Results and Discussion

### 3.1. Thermal Analysis

Table 1 summarizes the ΔH values obtained for the unfilled sample (acronym UF sample) and the sample filled with 3% by weight of as-received MWCNTs (acronym FR sample). The ΔH_dyn_ values in Table 1 have been taken as reference for the reaction enthalpy values, in order to evaluate the curing degree (by Equation (2)), for both systems, after the different curing cycles, in the oven and by the electro-curing process (Table 2).

The presence of carbon nanotubes caused only a slight decrease in the ΔH value during the dynamic scan. The curing process of a thermosetting composite is dependent on the nature of the resin, curing agent, filler, and their mutual interactions. Multi-stage curing cycles are recommended when careful and complete cross-linking reactions are requested to satisfy the requirements of high mechanical performant resins and, therefore, to fulfill industrial requirements. These cycles generally include a pre-curing stage at a low temperature and a post-curing stage at a higher temperature. In the pre-curing stage, small molecules are combined with larger units with a low degree of cross-linking (termed a B-stage resin). The post-curing stage allows the relaxation of the internal stresses formed during the cure reaction and makes the resin harder as a result of the cross-linking completion. In this work, several temperature cycles were analyzed to identify the optimal conditions for the investigated epoxy system. The properties of the samples cured with different curing cycles performed in the oven have been compared with the electro-cured samples. In Table 2, the tests performed using the DSC method are summarized along with the samples’ codes, the processing conditions, the total heat of reaction (ΔH_residual_) measured after the different curing cycles, and the reached curing degree. In particular, the samples cured in the oven through temperature cycles have been named with the initial acronym “FR”, whereas the electro-cured samples are named with the initial acronym “E”.

**Figure 3:**
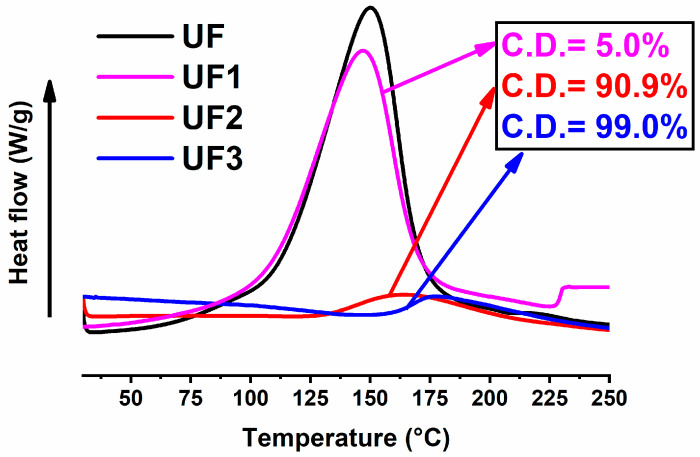
Dynamic differential scanning calorimetry (DSC) curing curves of the samples UF, UF1, UF2, and UF3.

Figure 3 shows the enthalpic curves recorded during a heating scan from 30 to 250 °C at 10 °C/min of the samples UF1, UF2, and UF3 after the curing cycles in the oven reported in Table 2. For comparison, the enthalpic curves of the samples after treatment in the oven have been compared with that of the sample without any thermal treatment (the fresh untreated formulation of Table 1).

**Figure 4:**
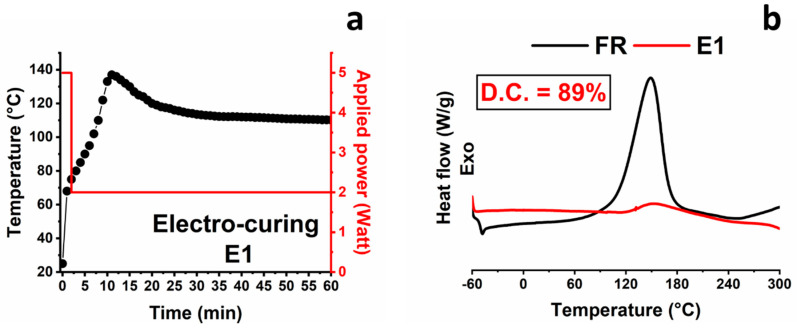
Electro-curing cycles for sample E1: (**a**) applied power and sample temperature vs. time detected for sample E1; (**b**) DSC curves of the samples FR and E1.

The values of the curing degree displayed in Figure 3 and in Table 2, for the different curing conditions and strategies, were obtained from equation 2 (see Section 2.1). From Figure 3, it is evident that for the UF sample series, high curing degrees are obtained for temperatures exceeding the value of 80 °C. For example, 20 min at 120 °C is necessary to reach about 91% of the full conversion (sample UF2). With an additional isothermal step at 180 °C for 60 min, a C.D. value of 99% of the full conversion is achieved (sample UF3). The filled sample FR3, subjected to the same curing cycle of sample UF3, manifested a lower C.D. with respect to the filled sample. This behavior is due to the presence of the nanofiller and has been already understood in the literature [31,45]. High initial temperatures do not guarantee a quicker and controlled cure. Tests have shown that it is better to perform a curing cycle composed of different steps, starting from a step at a lower temperature. In fact, as shown in Table 2, the sample FR4, subjected directly to an isothermal step at 180 °C for 180 min, reached a final C.D. of only 95%. As expected, faster and uncontrolled cross-linking reactions do not allow a systematic network formation and produce a high level of stress frozen in the solid samples [46]. At the end of this section, we will see that a very similar concept is also valid for the electro-curing process. The flow of an electric current through the material produces heat, due to the resistive nature of the epoxy/CNT nanocomposites, and, therefore, a temperature rise in the material. The increase in the temperature must be accurately controlled by adjusting the applied voltage and choosing electro-curing cycles suitable to obtain a good compromise between the material performance and the energy employed. Figure 4 concerns the sample E1, cured with an electro-curing stage composed of two steps—2 min@5 W + 58 min @2 W. In particular, the sample E1 was cured with an electric power step of 5 watts (corresponding to a voltage of 200 volts) for 2 min, followed by a power step of 2 watts (corresponding to a voltage of 136 volts) for 58 min.

**Figure 5:**
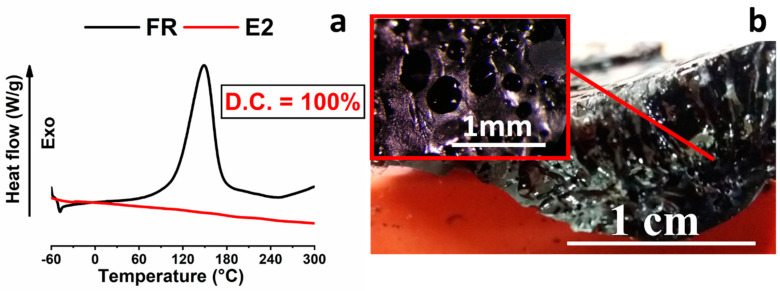
(**a**) DSC thermograms of the samples E2 (red trace) and FR (black trace); (**b**) optical image of the sample E2.

As shown in Figure 4a, the sample temperature quickly reaches the value of 70 °C during the first power step, and then, in the second step, a rapid increase culminating with a maximum at 137 °C occurs. After reaching the maximum value, the temperature slowly decreases to the final constant value of 110–112 °C. It is interesting to note that in the second step, despite the reduction in the applied power, the sample temperature initially continues to increase because of the autocatalytic nature of the cross-linking reaction. The isothermal plateau is reached after about 30 min and holds up to the end of the electro-curing stage. After that, the sample is allowed to cool at room temperature and analyzed by DSC analysis. In Figure 4b, the DSC trace of the filled sample E1 (red graph) shows that after the performed electro-curing stage, the sample is characterized by a D.C. value of about 89%. In Figure 4, the DSC curve of the reference sample (FR) is also shown for comparison. The D.C. value of 89% may result in not satisfying the mechanical performance necessary for several structural applications. With the aim to obtain a higher C.D. value, an electro-curing stage composed of steps performed at a higher power was performed. In particular, sample E2 was electro-cured with a stage composed of two electro-curing steps (5 min@10 W + 15 min@5 W). Figure 5a,b show the DSC curve, compared with the reference sample FR, and the optical image of the sample E2.

**Figure 6:**
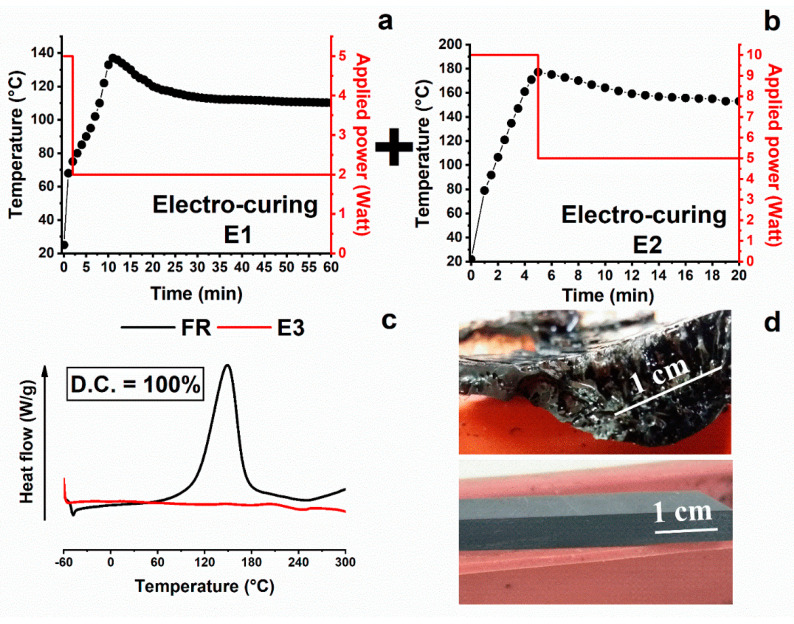
Electro-curing cycles carried out for obtaining sample E3: (**a**) applied power and sample temperature vs. time during the cycle electro-curing cycle E1; (**b**) applied power and sample temperature vs. time during the cycle electro-curing cycle E2; (**c**) DSC thermograms of the samples E3 (red trace) and FR (black trace); (**d**) optical images of sample E2 and sample E3.

Figure 5a highlights that after the complete electro-curing stage, sample E2 results completely cured (C.D. = 100), satisfying the previously fixed goal to reach a higher curing degree. Figure 5b shows the optical image of a cross-section of the sample. It evidences numerous trapped bubbles. Probably, the overheating during the electro-curing process leads to excessive gas production, which remains fixed during the quick solidification of the sample. The poor temperature control during the fast curing process also affects the final dimensions and shape of the sample, which become completely compromised. Therefore, although the goal to reach a high C.D. value, employing higher powers, was achieved, other critical issues had to be addressed. In consideration of all results herein described, two conclusions can be drawn. First, a single electro-curing stage performed employing higher values of the power, although giving rise to high C.D., induces an overheating of the sample, which negatively affects the morphology and then the dimensional stability of the sample. Most likely, in the case of the electro-curing process, as in the case of a conventional curing process performed in an oven or autoclave, inhomogeneities in the cross-linking density and local thermal degradations of the material are usually obtained if the sample is not cured with an electro-curing cycle composed of several stages. Second, the first electro-curing stage has to be performed by choosing steps suitable to reach lower values in the temperature of the sample, as usually occurs for samples traditionally cured in an oven or autoclave. The electro-curing stage chosen to obtain the sample E1 determines the reaching of a lower temperature with respect to that detected for the sample E2 electro-cured with an electro-curing stage composed of steps performed at higher power (not reported here), as it can be easily deduced for the presence of bubbles. To overcome the critical issues detected for sample E1 and sample E2, the electro-curing of the sample E3 was performed through a sequence of two stages. The first stage was carried out by reducing the power of the two planned steps, as performed for sample E1, and the second stage was carried out at higher powers, as performed for the sample E2. Then, sample E3 was cured with a two-stage electro-curing process composed of the first stage (2 min@5 W + 58 min@2 W) followed by the second stage (5 min@10 W + 15 min@5 W); therefore, sample E3 was solidified using the electro-curing sequence applied for sample E1 + sample E2. Figure 6 shows the temperature profile and the values of the applied power as a function of the time for sample E3 after the first stage (Figure 6a), which is the same as sample E1, and after the additional second electro-curing stage, indicated as E2 cycle (Figure 6b). It is worth noting that in this case, the electro-curing stage is the same as sample E2, but this cycle is carried out on the sample already electro-cured through the electro-curing cycle chosen for sample E1. The temperature profile and the values of the applied power as a function of the time for sample E3, after the first stage, were already discussed in the comments of Figure 4a. Figure 6b shows the temperature profile and the values of the applied power as a function of the time for sample E3 during the additional second electro-curing stage (indicated as E2 in the figure). The first step performed at a power of 10 watts (corresponding to a voltage of 280 volts) for 5 min determines an increase in the temperature up to ~ 180 °C. In the second step, which is performed by applying the reduced power of 5 watts (corresponding to a voltage of 205 volts), a slow decrease in the temperature is detected in the first 6 min and then the temperature reaches an almost constant value of ~160 °C.

Figure 6d shows the optical images of sample E2 cured with a single electro-curing stage (5 min@10 W + 15 min@5 W) and sample E3 cured with a two-stage electro-curing process ((2 min@5 W + 58 min@2 W) + (5 min@10 W + 15 min@5 W)). Although in both cases, the resin manifests a value of curing degree of 100%, the system obtained with a single-step power presents a non-compact structure due to the presence of entrapped bubble gas during the reaction. Sample E3 highlights good dimensional stability after the solidification through the two-stage electro-curing process. The morphological features at the micrometric scale of the cross-section of sample E3 will be discussed in the section “Morphological analysis”. It will highlight that the double curing stage limits the autocatalytic nature of the cross-linking reactions, thus avoiding the formation of bubbles and locally degraded regions near the electrode surface. Figure 6c, as shown in Table 2, evidences a complete C.D. (100%). The data in Table 2 also allow for making some considerations regarding the two filled and unfilled systems and the different curing processes. The electrical response to an external stimulus (e.g., electrical input) of the uncured filled matrix enables the production of heat for the Joule effect and the polymerization reaction of the material. However, tuning the electrical conductivity of the final conductive composite is not a trivial problem. According to the percolation theory [47,48,49], for each system, there is a minimum particle concentration required, the percolation threshold, to obtain a conductive particle network that enables the flow of electrical current. Further particle addition often brings a smaller improvement compared to the conductivity increase of several orders of magnitude that occurs around this threshold. For the filled samples, carbon nanotubes can be considered as a continuous network incorporated in the matrix. Their concentration is beyond the electric percolation threshold (EPT).

**Figure 7:**
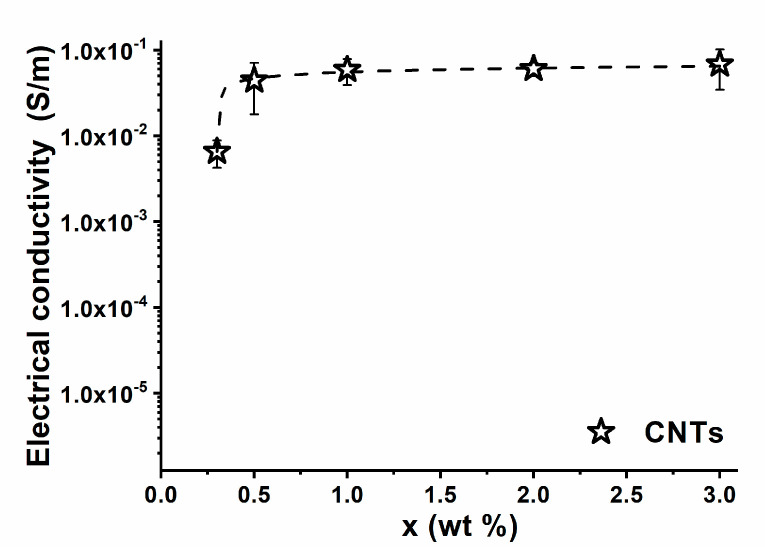
Electrical conductivity of the carbon nanotube (CNT)-based resin vs. filler weight percentage relating to samples cured by the curing cycle marked FR3 (curing cycle = 1 h@80 °C + 20 min@120 °C + 1 h@180 °C).

In fact, as shown in Figure 7, the electrical conductivity (σ) varies with the increase in filler loading (x), in agreement with the percolation theory according to a typical scaling law [47,48,49], for which an electrical electric percolation threshold (i.e., EPT) below 0.5 wt %, has been detected. This is indicative of the fact that the distances between carbon nanotubes are equivalent to the tunneling distance [32]. In light of these results, a concentration of 3% by weight of CNTs was chosen in order to maximize the electrical conductivity of the uncured sample with thermal conductivity, which is slightly affected by the nanofiller concentration [17,50]. A higher thermal conductivity allows for a more efficient heat transmission for the same applied voltage. CNTs can be considered as nanometric heating elements (as it was suggested in this work) or electrodes with high surface areas [33]. The heat generated by the electrical flow is then uniformly distributed throughout the matrix, minimizing, in this way, the thermal gradients in the sample. This results in a polymerization process intrinsically even more uniform than that detected for the sample cured in the oven. Proof of the greater effectiveness of the electro-curing process compared to the classical curing cycle in the oven is shown in Table 2. The C.D. of the electro-cured sample E1 is similar to that of the sample UF2, cured in the oven (89% and 91%, respectively). Sample UF2, in the final step of the curing process, was kept at the constant temperature of 120 °C for 20 min, while sample E1 reached the constant temperature of 110–112 °C after a maximum of 137 °C. Sample E3 reached a curing degree of 100%, with temperatures of 160 °C for a maximum of 20 min. Sample UF3 reached a C.D. of 99% after another hour at 180 °C, in addition to the curing cycle performed for sample UF2 (see Table 2). It is interesting to note that the filled resin, cured in the oven (sample FR3), when cured and also post-cured, reached a C.D. value of 91%, hence, a value lower than that of the unfilled resin cured in the same way (UF3). The difference in the C.D between samples UF3 and FR3 (99% and 91%, respectively) is probably due to the hindrance effect of the CNT network on the molecules cross-linking during the cure reaction [31,45]. This determines the creation, in the structure of the resin, of domains with lower curing degree. Sample E3 must be compared with sample FR3 (for the fact that it contains CNTs at the same percentage). It is interesting to observe that in the case of the electro-curing process, the C.D. is 100% with respect to the value of 91%, which is the maximum value obtained for the filled sample subjected to the more drastic curing cycle in the oven. A very interesting aspect detected for the electro-cured sample is that the highest curing degree was obtained with a less energy-intensive thermal process. In fact, the electro-curing process performed for sample E3, working in the range of power between 2 and 10 W, totally required 12.4 kJ to cure a sample having a volume of 50 × 25 × 8 mm^3^, with a weight of about 6.0 g. Performing a comparison with sample FR3, cured in the oven, with the same weight and dimensions, the power ranged between 350 and 850 W and the process required about 5 MJ for the curing of the sample (for which a C.D. of only 91% was achieved). Another benefit of electric heating is the possibility to better tailor the supplied electric power for the curing. This can result in even more favorable energy savings. As seen for the filled sample cured in the dynamic regime (see Figure 3), and for which the curing enthalpy is around 367 J/g, the exothermic reactions are activated in the initial temperature range between 60 and 80 °C. This temperature can be reached using a suitable electro-curing step, and in this case, it is possible to reduce the supplied electric power and to exploit the heating due to the polymerization reactions under the electrical treatment. The high thermal mass of an oven together with the low heating rates make this adjustment not feasible in an oven curing process.

### 3.2. Sorption and Mechanical Analysis

**Figure 8:**
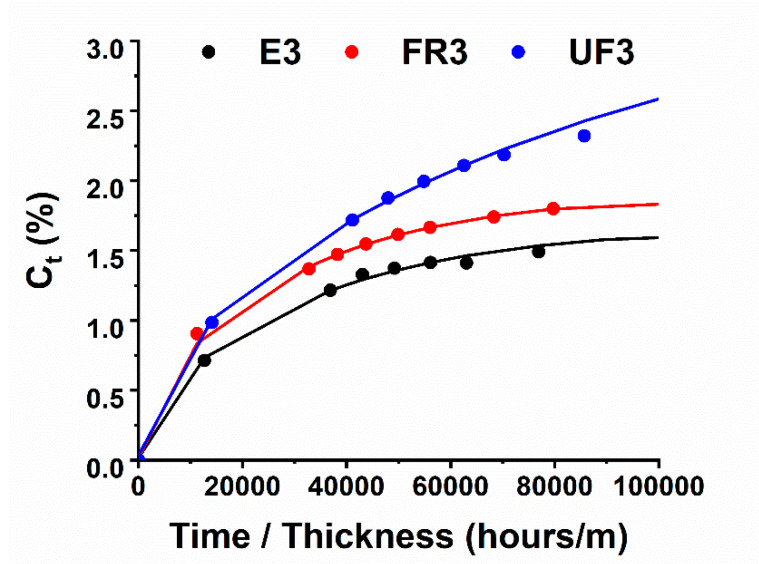
Water absorption measurements at 60 °C obtained with reported specimens. Lines are to guide the eye.

Figure 8 shows the variation in the percentage of water content in samples subjected to an absorption experiment at 60 °C for up to 15 days.

**Figure 9:**
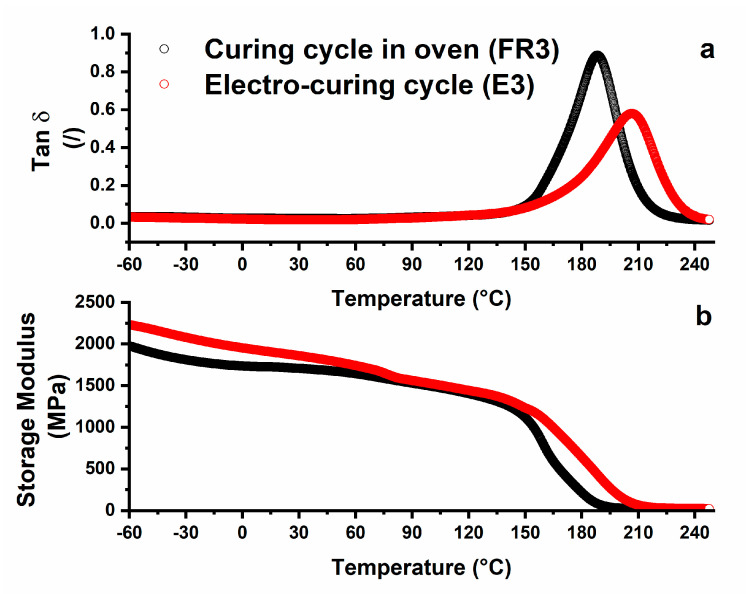
Dynamic mechanical analysis (DMA) relating to the composite after the different curing approaches: (**a**) loss factor (Tan δ); (**b**) storage modulus.

The sorption rate was very high during the first days of treatment. A higher sorption percentage is observed for the unfilled sample UF3, while the electro-cured sample shows a lower value. In literature, the water diffusion process in the epoxy matrix is well characterized. In particular, different transport stages are normally observed. The adsorbed water molecules first occupy the micro-cavities present in the samples; then, as the concentration of the diffusant increases, the water molecules bind to polymer chains (especially hydroxyls produced during curing reactions), causing material plasticization and swelling. The regions densely cross-linked are the last ones interested in water diffusion. The electro-cured sample is characterized by the highest percentage of dense cross-linking regions (as detected through DSC investigation), which highlights the intrinsic ability to be characterized by a reduction in the equilibrium adsorption of water molecules. The electro-curing process does not only allow a better diffusion of heat but also promotes some kinds of interaction between CNTs and the epoxy resin, determining a high curing degree. The dynamic mechanical analysis of the sample highlights this phenomenon. Different factors imposed during the curing process (e.g., heating procedure, time and frequency of mechanical stirring, and sonication) and the incorporation of fillers and/or small molecules, such as water, strongly affect the dynamic mechanical behavior of the resin [51,52]. Figure 9 shows a comparison between the evolution of the storage modulus and loss factor (i.e., Tan δ) for samples FR3 and E3, respectively. For both samples, the storage modulus (Figure 9b) is higher than 1600 MPa in a very wide temperature range (from −60 to 70 °C). After this temperature, a slow and progressive decrease up to 150 °C is observed, followed by a drop in the range from 180 to 200 °C. In the same temperature range, the loss factor curves show a peak, indicative of the polymer glass transition (Figure 9a), which is higher for the electro-cured sample, as expected, considering the higher cross-linking density. In addition, sample E3 shows a much more gradual decrease in the storage modulus in the region of the glass transition. In this sample, the higher degree of physical cross-links results in a higher storage modulus, as evident below the glass transition temperature. Tan δ is associated with longer-range cooperative molecular motion, which is consistent with the rubbery flow and permanent deformation. The shape and size of the peaks of Tan δ provide useful information on the relaxation phenomena of the material. It is known from the theory that the relaxation processes in polymers, and especially in composites, are complex mechanisms [53]. Several models have been proposed to describe these behaviors. Among others, the assumption that the whole process consists of discrete elementary relaxation steps represents a realistic and practical approach. Figure 9 shows that sample E3 manifests a much smaller peak in Tan δ. This means that the E3 sample has a much more elastic behavior with respect to the FR3 sample. The E3 sample also shows a peak with a greater width. A wider distribution of relaxation times, presumably due to the nanoparticle–polymer interactions and reduced mobility, is present in sample E3.

**Figure 10:**
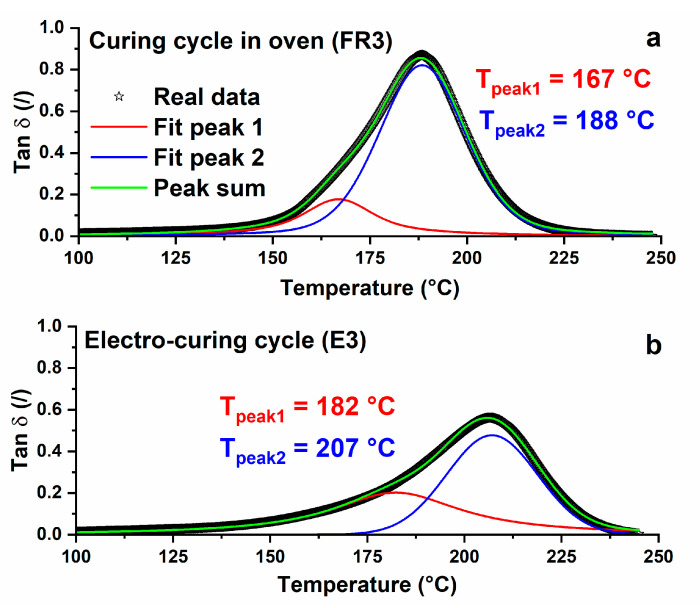
Master curve of the multiple α_i_-relaxations of the Tan δ relating to the composite after the different curing approaches: (**a**) sample FR3; (**b**) sample E3.

For both systems analyzed in Figure 9, Tan δ curves result from the superposition of two different peaks. The main one is at the higher temperature and a shoulder is placed at lower temperatures. At least two different relaxation mechanisms can be identified in the mechanical behavior of the composites. The principal intense mechanism is associated with the main structure formed by the reaction of the cross-linking agent with the epoxy groups. The less intense mechanism is probably due to the effect of the filler on the hardening reaction. As already reported in the literature, a strong resin–filler interface is expected to occur before the complete polymerization of the composite at a lower temperature [54,55,56]. This region, made of a layer a few nanometers-thick, induces the growth of the secondary peak (at about 167 °C, for the sample cured in the oven, and 182 °C, for the electro-cured sample), shown in Figure 10.

The glass transition process depends on the intrinsic flexibility of the polymer chain segments and on the free volume available within the polymer structure. The conformational change can only occur when there is sufficient free volume to allow movements of the chain segments. Changes in the chemical structure of the material, such as the formation of intermolecular cross-links, diminish the flexibility of the chain segments and determine an increase in the glass transition temperature of the material. Stimoniaris el al. [53] have suggested that a broad α-relaxation peak is the result of the superposition of multiple relaxations mechanism (α_i_), each with a characteristic transition temperature T_i_. In other words, the Tan δ versus temperature diagram can be considered as the result of a heterogeneous cross-linking topology. Figure 10 shows, for both curing procedure, a Tan δ profile, which can be approximated with two different peaks. The two peaks were calculated by applying a resolution algorithm based on the Levenberg–Marquardt method [57]. To reduce the number of adjustable parameters, the baseline and the peak equation were fixed. The peak function was a mixed Gauss–Lorentz equation [58]:

(4) $$f\left(x\right)=\;\left(1-L\right)Hexp\left[-4ln\left(2\right){\left(\frac{x-x_{0}}{w}\right)}^{2}\right]+LH{\left[4{\left(\frac{x-x_{0}}{w}\right)}^{2}+1\right]}^{-1}$$

where *x*_0_ = the peak position; *H* = peak height; *w* = FWHH (Full-Width Half Height); *L* = fraction of Lorentz character. In Figure 10, sample FR3 shows the peak 1 centered at about 167 °C and the peak 2 centered at the temperature of 188 °C. When the cure is done with a resistive cycle (E3), both peaks move at higher temperatures. More precisely, peak 1 moves to 182 °C and peak 2 to 207 °C. The resistive hardening cycle allows a more effective polymerization process both for the main structure, identified by the dominant α-relaxation, and for the resin–filler interface, identified by the lower temperature shoulder. The contribution of each reticulated structure to the formation of the overall behavior of the material depends on the characteristic weight of the two peaks; for this reason, it is possible to introduce an average value of the glass transition temperature [53]:

(5) $$T_{g\;av.}=\frac{\sum^{\;}T_{peak\;i}\times A_{i}}{\sum^{\;}A_{i}}$$

where *T_peak i_* is the temperature and *A_i_* is the area of the i-peak. The values obtained for both of the analyzed systems are more suitable in describing the complex α-relaxation profile. They reflect all the phenomena involved, contrary to glass transition values identified with the maximum of only the main peak of Tan δ. As it was easy to imagine, the resistive curing cycle provides a composite with a value of glass transition temperature (*T_g av_*) higher than that of the curing in the oven. More precisely, 186 and 200 °C for the curing cycle in the oven and the resistive curing cycle, respectively. The highest value of glass transition derives from a higher curing degree in the whole structure of the material, as confirmed by DSC analysis (see Table 2).

**Figure 11:**
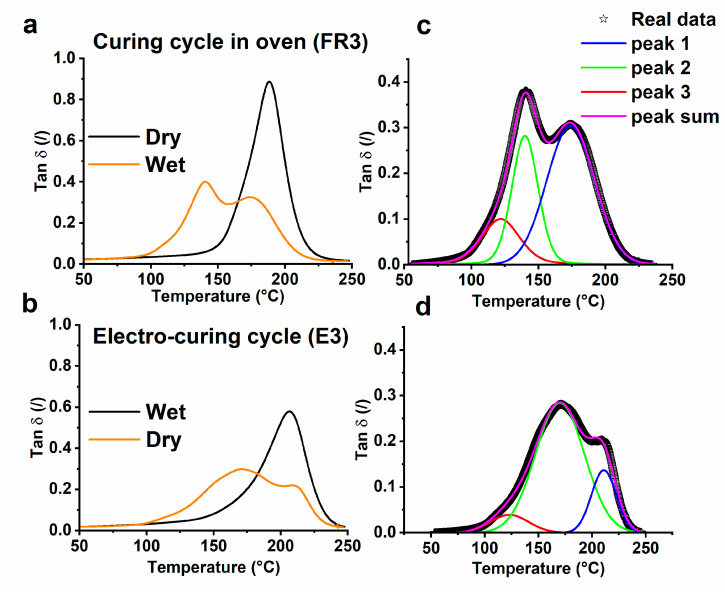
Tan δ relating to the composites before and after the water sorption experiments: (**a**) sample FR3 and (**b**) sample E3. Master curve of the multiple α_i_-relaxations of the Tan δ relating to the composite after water sorption: (**c**) sample FR3; (**d**) sample E3.

Figure 11 shows the effect of the water uptake on the loss factor (Tan δ) for the samples FR3 and E3. In both cases, the water molecules are able to produce a significant reduction both in the height of Tan δ profile and the value of *T_g_* with a consequent change in the profile and the peaks present. Moreover, it is clearly seen that a further relaxation peak occurs below *T_g_* for both systems. This peak of extra relaxation is attributed to the effect of water molecules bound to the polymeric segments, which, acting as a plasticizer or participating in the network by hydrogen bonds, influence the inter and molecular flexibility of the matrix and are responsible for introducing a new relaxation mechanism. The electro-curing process produces a polymer network containing very highly cross-linked regions as well as less interconnected, densely cross-linked regions. This results in high resistance to the absorption of water molecules, with a broad distribution of mobilities or relaxation times. More specifically, the average value of the glass transition temperature (*T_g av_*) is reduced from 186 to 155 °C for the FR3 sample and from 200 to 174 °C for the E3 sample. By comparing the above results, it is possible to conclude that the main cause of the observed differences in the peak position and curve profile is determined by the different curing procedures. Electrical conductivity measurements and morphological analyses were carried out to verify the final distribution of carbon nanotubes in the epoxy resin. The uncured composite is characterized by a resistance value of about 10 kΩ, which decreases to about 1 KΩ after curing. The decrease in resistance is the result of two different phenomena. The cure of the resin, with the relative development of intramolecular crosslinks, determines the reduction in the free volume, the increase in the density, and consequent shrinkage of the material. This causes a reduction in tunneling distances between the CNTs in the composite with a relative increase in the system’s electrical conductivity. Furthermore, the alignment of the carbon nanotubes induced by the application of the electric field can be responsible for a reduction in the electrical resistance of the composites [59]. Different approaches have been used to align the CNTs. Commonly used orientation methods include mechanical forces [60], magnetic fields [61], electric fields [62,63], and shear flow [64]. These approaches present limitations as mechanical and shear forces can damage the structure of CNTs [65], and magnetic fields are not suitable because a high magnetic field strength is required (>5 T) [65]. The alignment of CNTs by an electric field seems to be the most promising and versatile technique because it is suitable for a wide range of applications [66,67]. For instance, the application of an AC electric field of the order of 13 KV/m during the curing process of natural rubber-based nanocomposites allowed the formation of aligned conductive nanotube networks. The effects of MWCNTs anisotropy affect thermal conductivity, dielectric properties, and dynamic mechanical properties [68]. Another example of CNTs aligned in polymer systems was obtained by means of an AC electric field of the order of 7 KV/m [43]. In this work, the electro-cured samples were subjected to a DC electric field ranging between 2 and 6 kV/m. However, the electrical conductivity of the electro-cured samples, evaluated in the direction of the applied electric field, was 9.34 × 10^−2^ S/m, which is comparable to the value obtained for the sample cured in the oven (7.28 × 10^−2^ S/m). Probably, the concentration of CNTs used in our case (3%) is too high to allow an effective alignment of the nanotubes. Oliva-Aviles et al. have reported that for concentrations above 0.75%, it is not possible to appreciate differences between composites solidified with and without application of electric field [43]. It probably means that at high concentrations, the alignment of the CNTs, if present, does not contribute, in a relevant way, to the electrical properties of the composite.

### 3.3. Morphological Analysis

**Figure 12:**
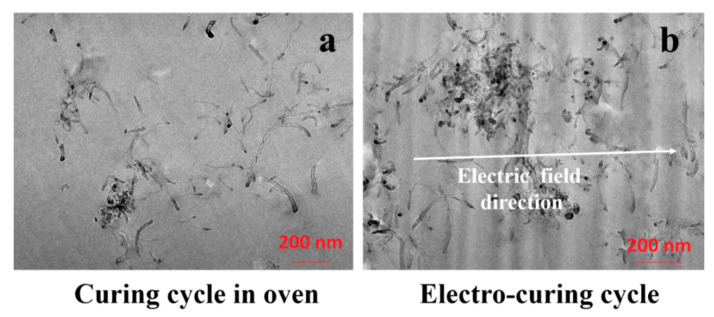
TEM images relating to the composite after the different curing approaches: (**a**) sample FR3; (**b**) sample E3.

The morphological analysis of the sample allows identifying the dispersion of the CNTs. Figure 12 shows the TEM images of samples FR3 (Figure 12a) and E3 (Figure 12b). To avoid possible confusion between the inevitable cut marks produced by the ultra-microtome and the orientation of the CNTs, the cutting direction was chosen to be orthogonal to the direction of the electric field.

**Figure 13:**
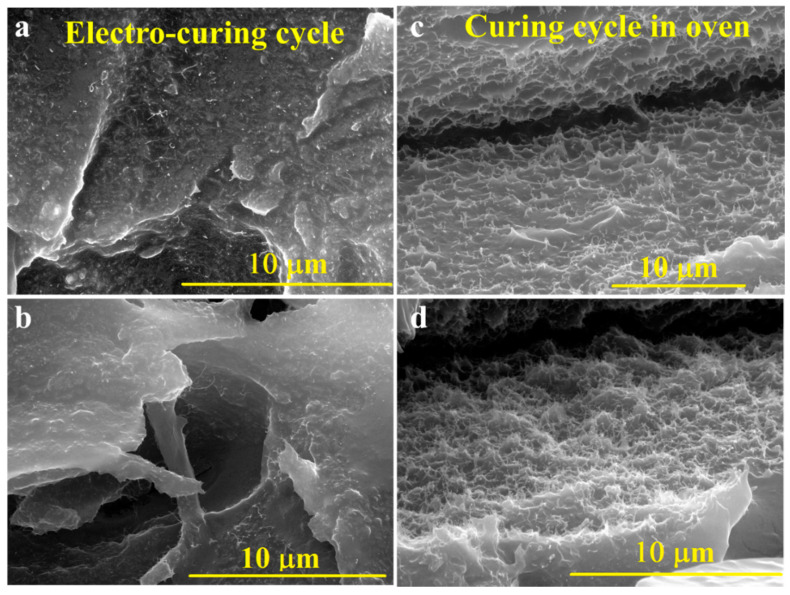
SEM images relating to the composite after the different curing approaches: (**a**,**b**) sample E3; (**c**,**d**) sample FR3.

In Figure 12b, the direction of the electric field is evidenced by a white arrow. When the film is obtained without the assistance of an electric field, individual CNTs look curled and randomly oriented. As expected, when the film is obtained without assistance from the electric field, the individual CNTs seem to be curled and oriented in a random way (Figure 12a). However, the electro-cured sample also does not show a preferential direction in the CNT orientation (Figure 12b). The concentration used in this work (3%) is well beyond the electrical percolation threshold of the system (<0.5%). In these conditions, the CNTs have reduced mobility, with a tendency to agglomerate during the mixing procedure [43]. Figure 13 shows the SEM images of samples E3 (Figure 13a,b) and FR3 (Figure 13c,d).

All the samples were treated with an etching solution before the SEM observations. A chemical attack was used to remove the epoxy resin, allowing, therefore, to better see the arrangement of the nanofiller in the matrix. Both systems present a homogeneous distribution of CNTs in the matrix. However, there is an important difference regarding the surface of the CNTs. In sample FR3, the CNTs appear almost completely naked; the etching process, although performed rigorously in the same way, completely removed the resin around the surface of the nanotubes, which rose from the matrix as thin wires (see Figure 13c,d). In sample E3, the resin sheathed the surface of the CNTs, which seem to be thicker and rougher (see Figure 13a,b). This is probably due to the higher resistance of the resin–filler interphase to the chemical attack [69]. This result confirms what was previously observed by DMA, DSC, and water absorption analysis. The highest glass transition temperature of the electro-cured sample (Figure 9) demonstrates the stronger interaction between the CNT surface and the epoxy resin. The higher degree of cross-linking reached accounts for the formation of links between the resin and the CNT surface.

## 4. Conclusions

In this work, the influence of the electro-curing process on the morphological features, mechanical and transport properties, and dimensional stability of an epoxy resin containing incorporated CNTs was analyzed. The performed investigation allowed for evaluating the differences between systems traditionally cured in an oven and a composite cured by electro-curing processes. In particular, electro-curing allows for obtaining shorter treatment cycles and higher curing degrees. The percentage of CNTs employed in this work does not allow the identification of eventual preferential orientations of the CNTs during the electro-curing. Furthermore, the electrical conductivity found was not very different between the two curing methods. In any case, the electro-cured sample shows=ed a higher value of glass transition temperature and a better interface between CNTs and the hosting epoxy matrix. These results suggest that electro-curing presents several advantages over other curing strategies. It is particularly advantageous for curing thick parts with a complex geometry. This process allows fine control over the cure degree and confers unique properties to the final material. The electro-curing method promises interesting applications in several fields, such as adhesives for structural joints and patches for the repairing of composites. In addition, it can also be used with traditional epoxy precursors when a higher T_g_ and a reduction in the equilibrium water sorption are required for specific applications.

## Figures and Tables

**Table 1:** Comparison of the ΔH values of the unfilled (UF) and filled (FR) samples.

Sample	Approach	Curing Cycle	ΔHdyn (J/g)
**UF**	DSC	Heating scan from 30 to 260 °C	375
**FR**	DSC	Heating scan from 30 to 260 °C	367

**Table 2:** Comparison of curing degree for the filled (FR and E) and the unfilled (UF) samples subjected to different curing approaches.

Sample	Method	Curing Cycle	ΔH_residual_ (J/g)	Curing Degree (C.D.) (%)
**UF1**	Oven	(1 h@80 °C)	356	5
**UF2**	Oven	(1 h@80 °C + 20 min@120 °C)	34	91
**UF3**	Oven	(1 h@80 °C + 20 min@120 °C + 1 h@180 °C)	4	99
**FR3**	Oven	(1 h@80 °C + 20 min@120 °C + 1 h@180 °C)	33	91
**FR4**	Oven	(3 h@180 °C)	10	95
**E1**	Electro-curing	(2 min@5 W + 58 min@2 W)	42	89
**E2**	Electro-curing	(5 min@10 W + 15 min@5 W)	0	100
**E3**	Electro-curing (steps performed on E1 + steps performed on E2)	(2 min@5 W + 58 min@2 W) + (5 min@10 W + 15 min@5 W)	0	100
